# The Effect of Whey Peptides and Micronutrients on Improving Exercise Performance in Mice

**DOI:** 10.3390/nu18020237

**Published:** 2026-01-12

**Authors:** Yitong Cheng, Chenxuan Wang, Jack Yang, Ziyue Wang, Haoran Xing, Wenbin Wu, Ting Yang, Hanfu Xian, Sitong Wan, Dongyuan Zhang, Na Li, Junjie Luo, Yongting Luo, Wanfeng Yang, Peng An

**Affiliations:** 1 Department of Nutrition and Health, Key Laboratory of Precision Nutrition and Food Quality, China Agricultural University, Beijing 100193, China; ccc666678@126.com (Y.C.); wangchenxuan0609@163.com (C.W.); wzy1234560820@163.com (Z.W.); xinghaoran2024@163.com (H.X.); wwb091828@163.com (W.W.); adeline7wan@163.com (S.W.); zhangdongyuan@cau.edu.cn (D.Z.); luojj@cau.edu.cn (J.L.); luo.yongting@cau.edu.cn (Y.L.); 2 Guangzhou SCA School, Guangzhou 510700, China; jack2018yhs@168.com; 3 Zhiyi Wellness Center for Healthy Living, Guangzhou 510180, China; sevenjasmine0904@168.com (T.Y.); xianhanfucn@163.com (H.X.); 4 Laboratory of Proteomics, Institute of Biophysics, Chinese Academy of Sciences, Beijing 100101, China; lina@ibp.ac.cn

**Keywords:** exercise training, muscle damage, anti-inflammatory, anti-fatigue

## Abstract

**Background**: Durative exercise-induced fatigue influences muscle structure and exercise performance. Dietary supplements combining bioavailable proteins with essential vitamins and minerals may help reduce fatigue. Compared with proteins, whey peptides, as easily absorbed energy sources, are regarded as better promoting the utilization of vitamins and minerals. This study investigated whether the combination of whey peptides and micronutrients could synergistically improve exercise-induced fatigue and exercise performance. **Methods**: Four-week-old male C57BL/6J mice were forced to exercise using a treadmill for four weeks to evaluate the supplemental effects of whey peptides and/or micronutrients on exercise performance. **Results**: Compared with mice receiving whey peptides or micronutrients alone, mice receiving a combination of whey peptides and micronutrients displayed increased muscle mass, muscle fiber cross-sectional area, muscle strength, and exercise performance, including running exhausting time and swimming exhausting time. Consistent results were obtained in detecting fatigue-associated serum metabolites and markers reflecting muscle injury. To elucidate the anti-fatigue mechanisms of whey peptides and micronutrients, RNA transcriptome in the muscle tissues were analyzed. Enrichment analysis results suggest that micronutrients and/or whey protein alleviate exercise-induced fatigue, not only via reducing oxidative stress but also repressing excessive immune activation in muscle tissue, thereby decreasing the tissue injury caused by strenuous exercise. **Conclusions:** Overall, the current study indicates that the combination of whey peptides and micronutrients produces a synergistic effect on promoting exercise performance. Our findings provide scientific evidence for the development of novel and efficient anti-fatigue functional foods using whey peptides and micronutrients.

## 1. Introduction

Endurance and strength training are fundamental for athletes to enhance exercise performance [1]. However, excessive exercise may induce chronic fatigue, a multifactorial phenomenon usually arising from energy depletion, oxidative stress, and muscle damage [2].

Exercise-induced fatigue is generally regarded as a reversible transient decline in physical performance. Several theories have been proposed to explain the underlying mechanisms, including exhaustion theory, clogging theory, and radical theory [3]. The exhaustion theory posits that the depletion of muscle and liver glycogen leads to fatigue [4]. The radical theory assumes that intensive exercise breaks the redox balance system, causing the accumulation of free radicals, causing body fatigue [5]. The clogging theory proposes that excessive accumulation of metabolites, such as blood lactic acid and blood urea nitrogen, causes metabolic disorders that eventually result in fatigue [6]. Aside from the physiological reaction to physical stress, fatigue may also arise from inflammation. Durative exercise generates pro-inflammatory cytokines, which may damage muscle structure and impair exercise performance [7,8]. Therefore, timely post-exercise nutritional supplementation targeting energy repletion, metabolite clearance, and antioxidant defense might constitute an effective multi-target intervention strategy against exercise-induced fatigue.

To mitigate exercise-induced fatigue while supporting daily training, sport nutrition supplements are increasingly sought because of their higher safety and fewer side effects [9,10]. Dietary supplements can better meet the nutritional requirements of the athletes with elevated physiological demands [11]. The International Olympic Committee Medical and Scientific Commission has recognized that certain dietary supplements can help athletes enhance exercise performance [12]. Meanwhile, a variety of evidence-based products, such as protein, vitamin, and mineral supplements, are available to promote muscle mass [13].

Protein supplements are widely used to support muscle development and performance [14,15]. Compared with proteins, peptides are an easily absorbed energy source which can more effectively promote the utilization of amino acids, as well as proteins and glucose [6]. In addition, peptides possess activities that may alter gut microbiota and improve body metabolism [16,17]. Whey peptides, a hydrolyzed form of whey protein, have been demonstrated to exhibit a higher absorption rate than proteins [18]. Numerous health benefits associated with whey peptides intake have been reported, including the antihypertensive, antioxidant, anti-obesity, anti-diabetic, and hypocholesterolemic effects [19,20]. Whey peptides have also been shown to accelerate recovery from exercise-induced muscle damage [21]. The underlying mechanism may be attributed to the mineral-binding capacity and vitamin protective capacities of whey peptides, which may improve the bioavailability of dietary micronutrients [22,23]. Adequate intake of micronutrients, such as minerals and vitamins, is crucial for exercise recovery because they are vital for energy metabolism, tissue repair, and overall well-being [24,25]. In addition, they play critical roles in various cellular processes, from ATP synthesis to redox balance maintenance [26]. Micronutrient-enriched supplements may positively enhance exercise capacity and delay the onset of fatigue through multiple pathways [15].

Micronutrient-enhanced beverages and functional foods are of great significance for promoting recovery from fatigue after intense training. However, the synergistic effects of micronutrients with whey peptides remain underexplored. This study investigates the anti-fatigue efficacy of a micronutrient formulation combined with whey peptides in exercise-trained mice by integrating physiological assessments (e.g., exhaustion time and oxidative markers) with analyses of underlying mechanism exploration. This multi-supplementation strategy is expected to produce superior, synergistic anti-fatigue effects mediated through multiple pathways.

## 2. Materials and Methods

### 2.1. Chemicals and Reagents

Vitamins and minerals, including sodium citrate, dicalcium phosphate, magnesium citrate, ferric pyrophosphate, calcium lactate, zinc gluconate, vitamin B_1_, vitamin B_2_, vitamin B_6_, vitamin B_12_, and vitamin C, were purchased from Hebei Pinkeyan Biotechnology Co., Ltd. (Baoding, China). Whey peptides (purity 98%) were purchased from Shanxi Baichuan Biotechnology Co., Ltd. (Jin’zhong, China).

### 2.2. Charaterication of Whey Peptides

Whey peptides were characterized through peptide enrichment and paper spray ionization mass spectrometry (PSI-MS) analysis. Peptide enrichment was performed using the EasyPept 96-well plate system (Omicsolution, Shanghai, China) according to the manufacturer’s protocol. Briefly, samples were dissolved in 0.1% (*v*/*v*) formic acid and loaded onto the C18 columns twice to ensure thorough binding of the peptides to the resin. Centrifugation at 700× *g* for 1 min was applied after each loading step to drive the sample through the resin and remove unbound contaminants. The columns were subsequently washed twice with 100 µL of D buffer and twice with 100 µL of E buffer. Enriched peptides were eluted with 40 µL of F buffer, and repeated three times, yielding a total eluate volume of 120 µL, which was used directly for subsequent mass spectrometric analysis.

For direct PSI-MS analysis, a triangular paper tip was prepared from Whatman 3MM chromatography paper (Cytiva, Maidstone, UK). The tip was mounted on a home-built PSI holder connected to a high-voltage power supply. For analysis, 10 µL of the peptide sample was spotted onto the center of the paper tip. The tip was positioned at a 30° angle, 5 mm from the inlet of a LTQ Orbitrap XL mass spectrometer (Thermo Fisher Scientific, Waltham, MA, USA). A spray voltage of +2.5 kV was applied to the brass holder, and the mass spectra were acquired in positive ion mode using a full scan FTMS method with the following settings: resolution 30,000, mass range *m*/*z* 200.00–4000.00, and maximum inject time 500 ms. Results are presented in the Supplemental Figure S1.

### 2.3. Animals

**Figure 1:**
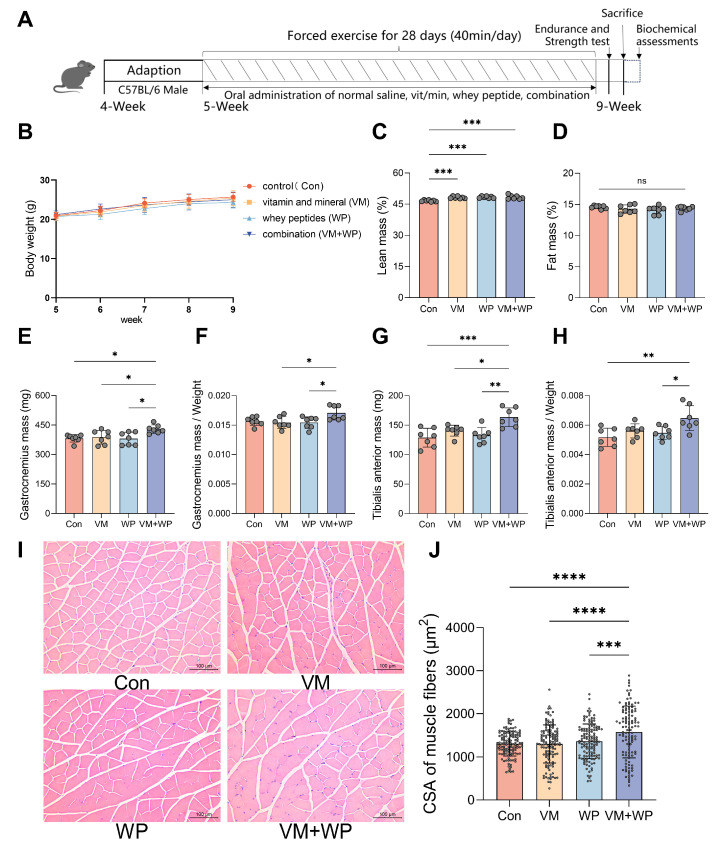
Effects of whey peptides and micronutrients on the body weight and muscle weight of mice forced to exercise (*n* = 7 in each group). (**A**) Experimental procedure. Mice were divided into four groups and subjected to forced exercise using a treadmill for four weeks. Simultaneously, four groups each received one of four daily dietary interventions with either saline (Con), micronutrient complex (vitamins and minerals, VM), whey peptides (WP), or micronutrient complex and WPs (VM + WP) during the four-week exercise. Then mice were tested for exercise tolerance and strength. (**B**) Body weight according to mouse age. (**C**) Lean mass. (**D**) Fat mass. (**E**) Gastrocnemius muscle (GAS) weights, and (**F**) the relative weights of GAS muscle. (**G**) Tibialis anterior muscle (TA) weights, and (**H**) the relative weights of TA muscle. (**I**) Histological examination of skeletal muscle tissue. (**J**) The cross-sectional area (CSA) of muscle fibers was quantified in three randomly selected mice from each group. Statistical significance was determined by one-way ANOVA with Duncan’s range test for multiple comparisons (* *p* < 0.05, ** *p* < 0.01, *** *p* < 0.001, **** *p* < 0.0001).

Four-week-old male C57BL/6J mice were purchased from SPF Biotechnology Co., Ltd. (Beijing, China). Mice were maintained under controlled temperature (22 ± 2 °C), humidity (45 ± 5%), and air flow conditions with a fixed 12 h light/12 h dark cycle. After one week of acclimatization, mice were randomly divided into four groups (seven mice in each group): a control group, a group receiving micronutrient complex containing multiple vitamins and minerals (VM), a group receiving whey peptides (WP), and a group receiving a combination of VMs and WPs (VM + WP). All interventions were given to mice via daily oral gavage for four weeks. For the control group, 0.2 mL saline was given. The VM group received 0.2 mL saline containing the following micronutrients: sodium citrate 0.5 mg, potassium chloride 0.19 mg, magnesium citrate 0.865 mg, ferric pyrophosphate 0.04 mg, calcium lactate 1.25 mg, zinc gluconate 0.06 mg, vitamin B_1_ 0.001 mg, vitamin B_2_ 0.001 mg, vitamin B_6_ 0.001 mg, vitamin B_12_ 0.00001 mg, and vitamin C 0.1 mg. Mice in the WP group were administered 0.2 mL of saline containing 1 mg whey peptides. For the VM + WP group, mice received 0.2 mL of saline containing both micronutrient complex and 1 mg whey peptides. The composition and dosage of the complex were primarily formulated based on the recommended daily intake levels and relevant national standards (e.g., GB 15266 [27] and GB 24154 [28]). A detailed experimental scheme is displayed in Figure 1A. During the four-week intervention period, the body weights of mice were recorded weekly. Behavioral indicators and bioassays were detected after four weeks intervention. The experimental protocols were approved by the Institutional Review Board of the Animal Experiment Ethics Committee, Chinese Agriculture University (Beijing, China) (No. AW50505202-5-03).

### 2.4. Sample Collection

All mice were euthanized under anesthesia using tribromoethanol (250 mg/kg). Whole blood was obtained via cardiac puncture. Blood samples were placed at room temperature for 2 h to clot. The samples were centrifuged at 1000 rpm for 10 min to separate serum for further analysis. Tissues from the skeletal muscles, including gastrocnemius (GAS) and tibialis anterior (TA), were collected after washing with phosphate-buffered saline (PBS). Separated serum samples were frozen in liquid nitrogen and stored at −80 °C for subsequent measurements.

### 2.5. Analysis of Body Composition

Mice body composition was measured by using a non-invasive compositional analysis and imaging system designed for small animals (QMR23-060h-I, Niumag, Shanghai, China). After being weighed, each mouse was placed into an animal holder and then measured. Lean mass and fat mass were measured three times for each mouse.

### 2.6. Forelimb Grip Strength Test

An electronic grip strength meter was utilized to detect the forelimb grip strength of mice (Beijing Zhongshidchuang Science and Technology Development Co., Ltd., Beijing, China). The sensor was set to the peak mode. When the front paws of the mice grasped the grip wire, the maximum grip was recorded. The test was repeated three times, with a brief rest of a few minutes between trials.

### 2.7. Animal Behavior of Hanging Test

In the hanging test, suspending mice based on their instinctive fear of falling evaluates grip endurance, offering an indication of muscle strength. Each mouse was placed at the center of a horizontal grid. When the mice instinctively grasped the grid, it was inverted, and the time until the mouse fell was recorded. Additionally, the mouse was placed on a thin bar, and the falling time was recorded.

### 2.8. Forced Treadmill Test

All mice were subjected to chronic forced exercise on a treadmill, which automatically recorded the number of electric shocks, running speed, and exercise time. In the first week, the instrument was set at a velocity of 10 m/min for mice adaptation. Then, from week two to five, mice were made to run daily at a velocity of 12, 15, and 20 m/min, for 40 min. In order to force the mice to run, the stimulation current of the treadmill was set at 0.5 mA.

After four weeks of training, the exercise tolerance test was performed. Mice were placed on the treadmill with a velocity of 20 m/min for 40 min. If the mice stopped running for 10 s, they were considered exhausted, and the exhaustion time was recorded. The number of electric shocks during the entire exercise over the three days prior to the exercise tolerance test was recorded to calculate the average of the electric shock number.

### 2.9. Forced Swimming Test

The forced swimming test was performed in a container filled with water (depth 20 cm). The water temperature was approximately 30 °C during the test sessions. Before evaluating the exercise endurance time, all mice received three days of swim training. Mice were considered to be exhausted when they failed to rise above the water surface in 10 s, and the swimming endurance time was recorded. After swimming, mice were dried with a towel.

### 2.10. Determination of the Organ Index

Various muscles were weighed using electronic scales. The formula of the organ index is shown below: Organ indexes (%) = Organ weight/Final body weight × 100%.

### 2.11. Detection of Serum Biochemical Parameters

To analyze fatigue-associated biochemical parameters, blood urea nitrogen (BUN), lactic acid (LA), and creatine kinase (CK) were measured by using commercial kits (Leidu/Changchun Huili, G4308, Beijing Solarbio Science and Technology Co., Ltd., Beijing, China). Malondialdehyde (MDA) was measured using commercial kits (G4302, G4306, Beijing Solarbio Science and Technology Co., Ltd., Beijing, China). Inflammatory cytokine TNF-α and IL-6 were assessed with enzyme-linked immunosorbent assays (TNF-alpha Mouse Uncoated ELISA Kit, and Mouse IL-6 ELISA Kit, Thermo Fisher Scientific). Determination and method of operation were performed according to the recommended procedures provided by the manufacturers.

### 2.12. Hematoxylin–Eosin (HE) Staining

Muscle tissues were fixed in 4% paraformaldehyde for 24 h and then embedded in paraffin. Tissues were cut into 4 μm thick slices for morphological and pathological evaluation. HE staining was performed using a commercial kit (G1120, Solarbio, Beijing, China). Morphological changes were observed using an optical microscope (CTR6, Leica, Wetzlar, Germany). The cross-sectional area of muscle fiber was analyzed according to a previous study [29] using Image J software (version 1.8.0, National Institutes of Health, Bethesda, MD, USA).

### 2.13. RNA Sequencing Analysis

Total RNA was extracted from muscle tissues using Trizol reagent (Thermo Fisher Scientific). Extracted RNA was then reverse-transcribed into complementary DNA using a commercial kit (HiScript III RT SuperMix, Vazyme, Nanjing, China) according to the manufacturer’s instructions. The sequencing library was prepared and sequenced using the Illumina hiSep sequencing platform. The clean reads were compared to the mouse reference genome using Hierarchical Indexing for Spliced Alignment of Transcripts. Gene and transcript expression levels were calculated using RNA-seq by Expectation Maximization (RSEM). Differentially expressed genes (DEGs) were screened and subjected to cluster and functional enrichment analysis. DEG detection was analyzed using the PoissonDis algorithm. DEGs with significant differences were assessed using false-discovery rate (FDR). DEGs were defined as genes with FDR ≤ 0.001 and a fold difference of 2 or more by default in this study. Graphic visualization was generated using the ggptot2 package in R software (v. 3.4.3). Enrichment analysis of DEGs was performed based on functional and biological pathways in Kyoto Encyclopedia of Genes and Genomes (KEGGs).

### 2.14. Statistical Analysis

Data are presented as means ± standard deviations. One-way analysis of variance and Duncan’s multiple range test were used for multiple comparisons. All *p* values are two-sided, and *p* < 0.05 was considered as statistically significant. GraphPad Prism 9 (v. 9.3.0, San Diego, CA, USA) was used for graph plotting and statistical analyses.

## 3. Results

### 3.1. Effects of Whey Peptides and Micronutrients on Body Weight, Body Composition, and Muscle Weight

Mice were divided into four groups and subjected to forced exercise using a treadmill for four weeks. Simultaneously, four groups received one of four daily dietary interventions, either with saline (Con), a micronutrient complex (vitamins and minerals, VM), whey peptides (WP), or a combination of micronutrient complex and whey peptides (VM + WP) during the four-week exercise period (Figure 1A).

During the entire experiment process, no significant differences were observed in body weight (Figure 1B) or food intake (Supplemental Figure S2) among groups. The VM, WP, and VM + WP groups all displayed greater lean mass than the control group (Figure 1C), while fat mass did not differ significantly among the groups (Figure 1D). The VM + WP group displayed increased skeletal muscle weights compared with the other groups, including increased GAS muscle weight (Figure 1E,F) and TA muscle weight (Figure 1G,H). These findings indicate that the combination of whey peptides and micronutrients exerts a synergistic effect on increasing muscle weights during exercise.

Histological examination of skeletal muscle tissue revealed no sign of lesion across all groups. Distinct morphological improvements in the muscle sections were found in mice receiving both micronutrients and whey peptides (VM + WP). As manifested in Figure 1I,J, muscle cells in the VM + WP group were enlarged, and interstitial components were reduced. Quantitative morphometric analysis confirmed these observations, showing that the cross-sectional area (CSA) of muscle fibers in the VM + WP group was significantly higher than that in the other three groups.

### 3.2. Effects of Whey Peptides and Micronutrients on Exercise Performance

**Figure 2:**
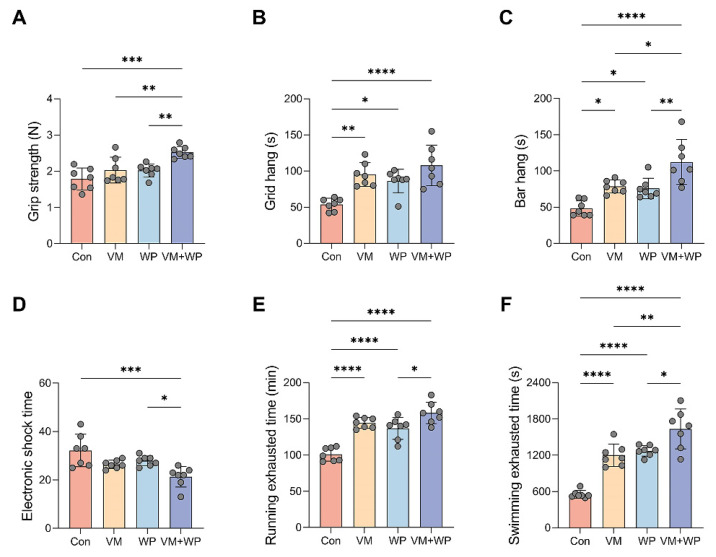
Effects of whey peptides and micronutrients on exercise performance (*n* = 7 in each group). (**A**) Grip strength, (**B**) grid hang time, (**C**) bar hang time, (**D**) electronic shock time, (**E**) running exhausting time, and (**F**) swimming exhausting time in mice receiving saline (Con), micronutrient complex (vitamins and minerals, VM), whey peptides (WP), or micronutrient complex and whey peptides (VM + WP). Statistical significance was determined by one-way ANOVA with Duncan’s range test for multiple comparisons (* *p* < 0.05, ** *p* < 0.01, *** *p* < 0.001, **** *p* < 0.0001).

Muscle functional capacity can be reflected by forelimb grip strength and tested by grid hanging and bar hanging. Following chronic forced-exercise training, animals in the VM + WP group demonstrated enhanced muscular performance compared with other groups. As shown in Figure 2, the VM + WP group exhibited not only greater forelimb grip strength (Figure 2A), but also markedly prolonged latency to fall in both grid and bar hanging tests (Figure 2B,C), indicating improved muscle endurance and fatigue resistance.

To evaluate the effects of whey peptides and micronutrients on mice exercise capacity, running and swimming exhaustion models were employed to assess their anti-fatigue efficacy [30]. Exercise capacity was substantially improved in the VM + WP group, as evidenced by a significant decrease in the average electric shock time (Figure 2D). Consistent results were obtained in the exhaustive running (Figure 2E) and swimming tests (Figure 2F). Greater endurance capacity in the VM + WP group directly reflects the fact that the combination of whey peptides and micronutrients generates synergistic anti-fatigue effects in mice subjected to chronic exercise load.

### 3.3. Effects of Whey Peptides and Micronutrients on Serum Markers Reflecting Exercise Performance

**Figure 3:**
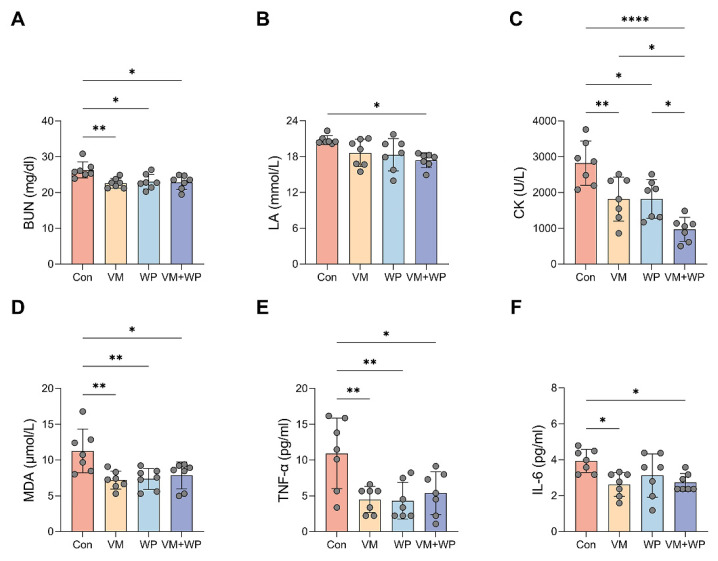
Effects of whey peptides and micronutrients on serum markers reflecting exercise performance (*n* = 7 in each group). In mice receiving saline (Con), micronutrient complex (vitamins and minerals, VM), whey peptides (WP), or micronutrient complex and whey peptides (VM + WP), the following serum parameters were measured: (**A**) Blood urea nitrogen (BUN), (**B**) lactate acid (LA), (**C**) creatine kinase (CK), (**D**) malondialdehyde (MDA), (**E**) tumor necrosis factor-α (TNF-α), and (**F**) interleukin-6 (IL-6). Statistical significance was determined by one-way ANOVA with Duncan’s range test for multiple comparisons (* *p* < 0.05, ** *p* < 0.01, **** *p* < 0.0001).

To further investigate the effect of whey peptides and micronutrients, fatigue-associated serum metabolites and markers reflecting muscle injury were measured. The combined supplementation of micronutrients and whey peptides (VM + WP) significantly lowered the serum levels of key fatigue-associated metabolites, including blood urea nitrogen (BUN, Figure 3A) and lactate acid (LA, Figure 3B), indicating a more efficient management of energy metabolism and a reduction in metabolic waste accumulation during exhaustive exercise. Concurrently, a pronounced decrease in creatine kinase (CK) activity was observed (Figure 3C), suggesting a significant mitigation of exercise-induced muscle fiber damage and improved membrane integrity. The VM + WP group also exhibited reduced lipid peroxidation product malondialdehyde (MDA, Figure 3D) and decreased inflammatory cytokines tumor necrosis factor-α (TNF-α, Figure 3E) and interleukin-6 (IL-6, Figure 3F). Collectively, the combined supplementation of micronutrients and whey peptides ameliorated exercise-induced stress via attenuating muscular damage, enhancing the body’s antioxidant capacity, and moderating the ensuing inflammatory response.

### 3.4. RNA Sequencing Analysis of Muscle Tissue and Enrichment Analysis

To further explore the underlying mechanisms, RNA sequencing analysis was performed on skeletal muscle tissues from mice in the four groups (three mice in each group). Differential expressed genes (DEGs) were identified among the groups (Supplemental Figure S3). Compared with the control group, the VM group showed 60 upregulated genes and 180 downregulated genes, while the WP group showed 232 upregulated genes and 128 downregulated genes. The VM + WP group showed 20 upregulated genes and 367 downregulated genes compared with the control group.

**Figure 4:**
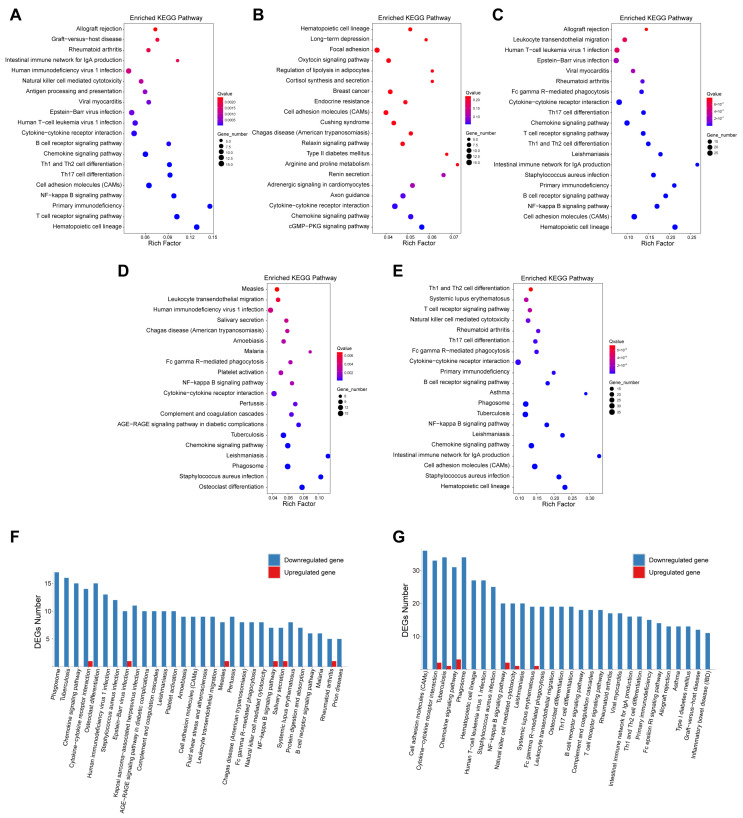
RNA sequencing analysis of mice muscle tissue and enrichment analysis. RNA sequencing analysis was performed in skeletal muscle tissues from mice in four groups (three mice in each group). Kyoto Encyclopedia of Genes and Genomes (KEGGs) pathway enrichment analysis was performed using differential expressed genes (DEGs) identified among groups. Enriched pathways using DEGs comparing VM group (**A**), WP group (**B**), or VM + WP group (**C**) with control group. Enriched pathways using DEGs comparing VM + WP group with VM group (**D**) or WP group (**E**). The up- and downregulated DEG numbers in enriched pathways by comparing VM + WP group with VM group (**F**) or WP group (**G**).

Compared with the control group, Kyoto Encyclopedia of Genes and Genomes (KEGGs) pathway analysis revealed that DEGs in both the VM group and the VM + WP group were enriched in hematopoietic cell lineage, the NF−kappa B signaling pathway, and the B cell receptor signaling pathway, whereas the chemokine signaling pathway was enriched in both the VM group and the WP group (Figure 4A–C). Additionally, DEGs were enriched in the phagosome, chemokine signaling pathway, cytokine–cytokine receptor interaction, NF–kappa B signaling pathway, and Fc gamma R-mediated phagocytosis pathways when comparing the VM + WP group with the VM group or the WP group (Figure 4D,E). Notably, most genes in these immunoregulatory pathways exhibited a downregulation trend (Figure 4F,G) [31]. This systematic downregulation of immunoregulatory pathways suggests that the combination of whey peptides and micronutrients effectively attenuates the exercise-induced overactivation of inflammatory responses.

## 4. Discussion

### 4.1. The Main Finding of the Study and Its Comparison with the Previously Published Literature

In recent years, increasing emphasis on improving exercise performance has driven the rapid growth of micronutrient supplements. These supplements promote the accumulation of specific nutrients within skeletal muscle beyond what can be achieved through ordinary dietary intake, thereby facilitating a more efficient exercise recovery [32]. Multivitamin and mineral regimens have been demonstrated to support athletic performance and enhance training adaptation [33,34]. For example, B vitamins function as cofactors in energy metabolism processes, and supplementation with vitamins B_1_, B_2_, B_6_, and B_12_ has been shown to improve exercise performance [35]. Vitamin C supplementation attenuates oxidative stress and the inflammatory response [36]. Iron supplementation improves oxygen transport and aerobic capacity via promoting hemoglobin synthesis [37]. Magnesium and zinc contribute to muscle energetic metabolism and muscle repair [38,39]. Calcium plays a crucial role in promoting neuromuscular transmission and muscle contraction efficiency [40]. Electrolytes such as sodium and potassium maintain fluid balance and ensure proper cellular function during exercise [41].

Whey peptides, which possess antioxidant and anti-inflammatory properties, have been reported to enhance exercise performance and accelerate post-exercise recovery [42]. For example, incorporating whey peptides to an antioxidant beverage was shown to further augment the anti-inflammatory effect of the beverage [43]. This synergistic effect of bioactive peptides and micronutrients presents a promising approach for comprehensive exercise support.

To provide scientific evidence for the development of new products with anti-fatigue potential, we evaluated the synergistic anti-fatigue efficacy of micronutrients with whey peptides. In this study, our findings support our hypothesis that whey peptides and micronutrients yield additive effects on improving exercise performance. On one hand, the combination of whey peptides and micronutrients increased lean body mass content without changing overall body weight (Figure 1); on the other hand, muscle strength, a direct indicator of exercise performance, was also enhanced in mice receiving both whey peptides and micronutrients (Figure 2A–C). Histological examination of skeletal muscle revealed an improved muscle structure in mice receiving both whey peptides and micronutrients, characterized by increased myofiber cross-sectional area and reduced interstitial components (Figure 1I). These structural adaptations are consistent with enhanced fatigue resistance and provide a histological basis for the observed functional improvements. Results from running and swimming exhaustion tests also clearly showed that the combination of whey peptides and micronutrients exerts a synergistic anti-fatigue effect by elevating muscle strength compared with mice receiving micronutrients or whey peptides alone (Figure 2D–F).

Beyond exercise behavior, the anti-fatigue effect of whey peptides and micronutrients was further evaluated using fatigue-associated metabolites and markers reflecting muscle injury, including BUN, LA, and CK in the serum. BUN is a metabolite of protein and amino acids and is an important indicator for estimating fatigue [44]. Ammonia produced by amino acid metabolism can reduce endurance and cause fatigue. BUN levels in the VM + WP group were reduced, indicating the enhancement of endurance (Figure 3A). LA as the main energy source during intense exercise in a short time can indicate the degree of fatigue. It is a glycolysis product of carbohydrates under anaerobic conditions. Decreased accumulation of serum LA means the improvement of energy metabolism and less fatigue [45]. A comparable reduction result with BUN was observed in serum LA levels (Figure 3B). CK is an enzyme that usually exists in skeletal muscle. One function of cellular CK is to add a phosphate group to creatine to synthesize the high-energy phosphocreatine molecule, which can be utilized as a rapid source of energy by the cells. Serum CK is well known to be an accurate indicator of tissue damage, and increased serum CK represents the fact that muscle damage has occurred [5]. There is a positive correlation between the serum CK and exercise tolerance [46]. Our results showed that the VM + WP group had lower serum CK levels than the VM or WP group (Figure 3C), which means the combination of micronutrients and whey peptides has a superior effect on preventing muscle damage than micronutrients or whey peptides alone.

MDA is a product of lipid peroxidation and serves as an indicator of oxidative stress in cells and tissues [47]. Elevated MDA indicates increased oxidative damage occurred in the cell membrane. Intense exercise also leads to the excessive release of pro-inflammatory cytokines such as TNF-α and IL-6. These cytokines are predominantly produced by monocytes and macrophages, and both of them could exert adverse effects on exercise performance and increase the sensation of fatigue during exercise [48,49]. In this study, decreases in MDA, TNF-α, and IL-6 were found in mice receiving whey peptides and/or micronutrients, indicating that these interventions improve exercise performance by reducing oxidative stress and inflammatory responses.

To further elucidate the anti-fatigue mechanisms of whey peptides and micronutrients, RNA transcriptomes in muscle tissues were analyzed. RNA-seq results indicated that DEGs in all intervention groups were significantly enriched in the chemokine signaling pathway, cytokine–cytokine receptor interaction, cell adhesion molecules (CAMs), and hematopoietic cell lineage, indicating that multiple inflammatory responses are involved during intense exercise. Notably, most DEGs in these signaling pathways were downregulated, suggesting anti-inflammatory effects in mice receiving micronutrients and/or whey protein interventions. Furthermore, DEGs from VM + WP/VM or VM + WP/WP comparisons revealed enrichment in leukocyte transendothelial migration, the NF–kappa B signaling pathway, and Fc gamma R-mediated phagocytosis (Figure 4D,E). Research has demonstrated that the anti-fatigue effect could be achieved via blocking the NF–kappa B signaling pathway to inhibit inflammation responses [50]. Meanwhile, our transcriptomic profiling further demonstrated a distinct downregulation trend across most immune system pathways in muscle from mice receiving both micronutrients and whey peptides, providing mechanistic insight into their synergistic anti-fatigue effect (Figure 4F,G). Our findings suggest that micronutrients and/or whey peptides alleviate exercise-induced fatigue, not only via reducing oxidative stress but also by suppressing excessive immune activation, thereby mitigating tissue injury caused by strenuous exercise.

### 4.2. The Immediate and Future Clinical Implication of the Findings in the Study

Overall, supplementation with a combination of whey peptides and micronutrients has the potential ability to serve as a useful nutritional aid for improving training quality and exercise performance, based on the dosing strategy satisfied with relevant standards. It also has translational potential for the development of targeted nutritional products aimed at preventing injury before exercise and improving recovery after training, particularly in adolescent and young adult athletes, who undergo rapid growth and development and have increased nutritional needs.

### 4.3. The Limitations of This Study

Despite these positive findings, this study has several limitations that warrant consideration. First, the use of 4-week-old mice limits comparability to adult or athletic human populations due to differences in hormonal profiles, muscle adaptation, and recovery capacity. Second, the treadmill test employed a mild electrical stimulus to encourage running, which may have added extra stress and affected test specificity. Third, although the sample size (*n* = 7 per group) is consistent with similar preclinical exercise studies, it may not have been sufficient to promote on a larger and broader scale.

### 4.4. The New Directions for Future Research

Based on these considerations, future research should aim to achieve the following: (1) include adult animal models and human cohorts to confirm broader applicability across different age groups; (2) employ modern protocols that favor non-painful motivational methods such as compressed air or visual stimuli, to ensure animal welfare and experimental specificity; and (3) increase the sample size to strengthen conclusions and potentially reveal more subtle phenotypic differences.

## 5. Conclusions

The combination of whey peptides and micronutrients produces a synergistic anti-fatigue effect by elevating exercise performance, decreasing fatigue-related biomarker levels, increasing fiber cross-sectional area, and suppressing the expression of genes in immune-inflammatory pathways. These findings provide a scientific foundation for developing novel and efficient anti-fatigue functional foods containing whey peptides and micronutrients. Further research is needed to focus on the clinical translation in human athletes to validate their efficacy.

## Data Availability

The original contributions presented in this study are included in the article/Supplementary Materials; further inquiries can be directed to the corresponding authors.

## Supplementary Materials

The following supporting information can be downloaded at https://www.mdpi.com/article/10.3390/nu18020237/s1, Figure S1: Paper spray ionization mass spectrometry (PSI-MS) analysis of whey peptides; Figure S2: Food intake in mice receiving whey peptides and/or micronutrients; Figure S3: Differential expressed genes in skeletal muscle tissues from mice in four groups (three mice in each group).

## Abbreviations

The following abbreviations are used in this manuscript:

BUN	Blood urea nitrogen
CK	Creatine kinase
CSA	Cross-sectional area
DEGs	Differentially expressed genes
FDR	False-discovery rate
GAS	Gastrocnemius muscle
HE	Hematoxylin–Eosin
IL-6	Interleukin-6
KEGGs	Kyoto Encyclopedia of Genes and Genomes
LA	Lactic acid
MDA	Malondialdehyde
PSI-MS	Paper spray ionization mass spectrometry
TA	Tibialis anterior
TNF-α	Tumor necrosis factor-α

## References

[1] Hughes D.C., Ellefsen S., Baar K. (2018). Adaptations to Endurance and Strength Training. Cold Spring Harb. Perspect. Med..

[2] Ma X., Chen H., Cao L.X., Zhao S., Zhao C., Yin S.T., Hu H.B. (2021). Mechanisms of Physical Fatigue and its Applications in Nutritional Interventions. J. Agric. Food Chem..

[3] Nan J., Li J.L., Wu H.S., Cheng H.R., Park H.J., Zhao Q.S., Yang L. (2024). Anti-fatigue activity and mechanism of crocetin loaded nanoliposome in acute exercise-treated mice. Food Sci. Hum. Wellness.

[4] Chen Y.J., Baskaran R., Shibu M.A., Lin W.T. (2022). Anti-Fatigue and Exercise Performance Improvement Effect of Glossogyne tenuifolia Extract in Mice. Nutrients.

[5] Luo C.H., Xu X.R., Wei X.C., Feng W.W., Huang H.Z., Liu H.Y., Xu R.C., Lin J.Z., Han L., Zhang D.K. (2019). Natural medicines for the treatment of fatigue: Bioactive components, pharmacology, and mechanisms. Pharmacol. Res..

[6] Li Z., Wu F., Shao H., Zhang Y., Fan A., Li F. (2017). Does the Fragrance of Essential Oils Alleviate the Fatigue Induced by Exercise? A Biochemical Indicator Test in Rats. Evid. Based Complement. Altern. Med..

[7] Suzuki K., Tominaga T., Ruhee R.T., Ma S. (2020). Characterization and Modulation of Systemic Inflammatory Response to Exhaustive Exercise in Relation to Oxidative Stress. Antioxidants.

[8] Taherkhani S., Suzuki K., Castell L. (2020). A Short Overview of Changes in Inflammatory Cytokines and Oxidative Stress in Response to Physical Activity and Antioxidant Supplementation. Antioxidants.

[9] Chen Y., Wang J.J., Jing Z.H., Ordovas J.M., Wang J., Shen L.R. (2022). Anti-fatigue and anti-oxidant effects of curcumin supplementation in exhaustive swimming mice via Nrf2/Keap1 signal pathway. Curr. Res. Food Sci..

[10] González-Weller D., Paz-Montelongo S., Bethencourt-Barbuzano E., Niebla-Canelo D., Alejandro-Vega S., Gutiérrez Á.J., Hardisson A., Carrascosa C., Rubio C. (2023). Proteins and Minerals in Whey Protein Supplements. Foods.

[11] Zhang H.X., Wang R.W., Guo S.S., Tian Q.Q., Zhang S., Guo L., Liu T.M., Wang R. (2023). Lower serum magnesium concentration and higher 24-h urinary magnesium excretion despite higher dietary magnesium intake in athletes: A systematic review and meta-analysis. Food Sci. Hum. Wellness.

[12] Maughan R.J., Burke L.M., Dvorak J., Larson-Meyer D.E., Peeling P., Phillips S.M., Rawson E.S., Walsh N.P., Garthe I., Geyer H. (2018). IOC consensus statement: Dietary supplements and the high-performance athlete. Br. J. Sports Med..

[13] Hsu Y.J., Jhang W.L., Lee M.C., Bat-Otgon B., Narantungalag E., Huang C.C. (2021). Lactose-riched Mongolian mare’s milk improves physical fatigue and exercise performance in mice. Int. J. Med. Sci..

[14] Nieto Á.V.A., Diaz A.H., Hernández M. (2025). Are there Effective Vegan-Friendly Supplements for Optimizing Health and Sports Performance? a Narrative Review. Curr. Nutr. Rep..

[15] Brauwers B., Machado F.V.C., Beijers R., Spruit M.A., Franssen F.M.E. (2023). Combined Exercise Training and Nutritional Interventions or Pharmacological Treatments to Improve Exercise Capacity and Body Composition in Chronic Obstructive Pulmonary Disease: A Narrative Review. Nutrients.

[16] Cai J., Tao Y., Xing L., Zhang J., Wang Z., Zhu Z., Zhang W. (2024). Studying Antifatigue Mechanism of Tyr-Pro-Leu-Pro in Exercise Mice Using Label-Free Proteomics. J. Agric Food Chem..

[17] Feng Z., Wei Y., Xu Y., Zhang R., Li M., Qin H., Gu R., Cai M. (2022). The anti-fatigue activity of corn peptides and their effect on gut bacteria. J. Sci. Food Agric..

[18] Lee J.A., Shin M.R., Kim M., Kim H.Y., Choi H.Y., Seo Y., Choi H., Roh S.S. (2024). Whey Peptide Alleviates Muscle Atrophy by Strongly Regulating Myocyte Differentiation in Mice. Medicina.

[19] Madadlou A., Abbaspourrad A. (2018). Bioactive whey peptide particles: An emerging class of nutraceutical carriers. Crit. Rev. Food Sci. Nutr..

[20] Lim G., Lim Y. (2022). Effects of Whey Peptide Supplementation on Sarcopenic Obesity in High-Fat Diet-Fed Mice. Nutrients.

[21] Liu J., Wang X., Zhao Z. (2014). Effect of whey protein hydrolysates with different molecular weight on fatigue induced by swimming exercise in mice. J. Sci. Food Agric..

[22] Olvera-Rosales L.B., Cruz-Guerrero A.E., García-Garibay J.M., Gómez-Ruíz L.C., Contreras-López E., Guzmán-Rodríguez F., González-Olivares L.G. (2023). Bioactive peptides of whey: Obtaining, activity, mechanism of action, and further applications. Crit. Rev. Food Sci. Nutr..

[23] Guo Y., Wang F., Yang T., Li S., Dong J., Fan Y., Zhang Z., Zhao X., Hou H. (2024). Enhancement of vitamin B stability with the protection of whey protein and their interaction mechanisms. Food Chem..

[24] Tardy A.L., Pouteau E., Marquez D., Yilmaz C., Scholey A. (2020). Vitamins and Minerals for Energy, Fatigue and Cognition: A Narrative Review of the Biochemical and Clinical Evidence. Nutrients.

[25] An P., Wan S.T., Wang L.R., Xu T.C., Xu T., Wang Y.H., Liu J., Li K.J., Wang X.F., He J.J. (2024). Modifiers of the Effects of Vitamin D Supplementation on Cardiometabolic Risk Factors: A Systematic Review and Meta-Analysis. Engineering.

[26] Killilea D.W., Killilea A.N. (2022). Mineral requirements for mitochondrial function: A connection to redox balance and cellular differentiation. Free. Radic. Biol. Med..

[27] (2009). Sports Beverage.

[28] (2015). National Food Safety Standard—General Standard for Sports Nutrition Food.

[29] Wu W.B., Guo X.L., Qu T.Q., Huang Y.J., Tao J., He J., Wang X.P., Luo J.J., An P., Zhu Y.H. (2024). The Combination of Lactoferrin and Creatine Ameliorates Muscle Decay in a Sarcopenia Murine Model. Nutrients.

[30] Xie Q., Sun Y., Cao L., Chen L., Chen J., Cheng X., Wang C. (2020). Antifatigue and antihypoxia activities of oligosaccharides and polysaccharides from Codonopsis pilosula in mice. Food Funct..

[31] Al Mahmud A., Shafayet Ahmed S., Karim M.R., Al-Mamun M.R., Akhter S., Sohel M., Hasan M., Bellah S.F., Amin M.N. (2023). Clinically proven natural products, vitamins and mineral in boosting up immunity: A comprehensive review. Heliyon.

[32] Cai B.N., Yi X.X., Wang Z., Zhao X.T., Duan A.L., Chen H., Wan P., Chen D.K., Huang J.T., Pan J.Y. (2023). Anti-fatigue effects and mechanism of Syngnathus schlegeli peptides supplementation on exercise-fatigued mice. J. Funct. Foods.

[33] Beck K.L., von Hurst P.R., O’Brien W.J., Badenhorst C.E. (2021). Micronutrients and athletic performance: A review. Food Chem. Toxicol..

[34] Ayaz A., Zaman W., Radák Z., Gu Y. (2024). Green strength: The role of micronutrients in plant-based diets for athletic performance enhancement. Heliyon.

[35] Lee M.C., Hsu Y.J., Shen S.Y., Ho C.S., Huang C.C. (2023). A functional evaluation of anti-fatigue and exercise performance improvement following vitamin B complex supplementation in healthy humans, a randomized double-blind trial. Int. J. Med. Sci..

[36] Righi N.C., Schuch F.B., De Nardi A.T., Pippi C.M., Righi G.D., Puntel G.O., da Silva A.M.V., Signori L.U. (2020). Effects of vitamin C on oxidative stress, inflammation, muscle soreness, and strength following acute exercise: Meta-analyses of randomized clinical trials. Eur. J. Nutr..

[37] Zhou L., Mozaffaritabar S., Kolonics A., Kawamura T., Koike A., Kéringer J., Gu Y.D., Karabanov R., Radák Z. (2024). Long-term iron supplementation combined with vitamin B6 enhances maximal oxygen uptake and promotes skeletal muscle-specific mitochondrial biogenesis in rats. Front. Nutr..

[38] Zhang Y.J., Xun P.C., Wang R., Mao L.J., He K. (2017). Can Magnesium Enhance Exercise Performance?. Nutrients.

[39] Chu A., Petocz P., Samman S. (2017). Plasma/Serum Zinc Status During Aerobic Exercise Recovery: A Systematic Review and Meta-Analysis. Sports Med..

[40] Peeling P., Sim M., McKay A.K.A. (2023). Considerations for the Consumption of Vitamin and Mineral Supplements in Athlete Populations. Sports Med..

[41] Heffernan S.M., Horner K., De Vito G., Conway G.E. (2019). The Role of Mineral and Trace Element Supplementation in Exercise and Athletic Performance: A Systematic Review. Nutrients.

[42] Pan D., Zhang Z., Liu N., Ashaolu T.J. (2025). Whey Protein Nutrition in Sports: Action Mechanisms and Gaps in Research. Curr. Nutr. Rep..

[43] Hillman A.R., Taylor B.C.R., Thompkins D. (2017). The effects of tart cherry juice with whey protein on the signs and symptoms of exercise-induced muscle damage following plyometric exercise. J. Funct. Foods.

[44] Gomez-Cabrera M.C., Carretero A., Millan-Domingo F., Garcia-Dominguez E., Correas A.G., Olaso-Gonzalez G., Viña J. (2021). Redox-related biomarkers in physical exercise. Redox Biol..

[45] Cheng C., Zhang S., Chen C., Gong Y., Ding K., Li G., Jiang W., Zhang Z., He B., Hu Z. (2025). Cordycepin combined with antioxidant effects improves fatigue caused by excessive exercise. Sci. Rep..

[46] Xu C., Lv J.L., Lo Y.M., Cui S.W., Hu X.Z., Fan M.T. (2013). Effects of oat β-glucan on endurance exercise and its anti-fatigue properties in trained rats. Carbohydr. Polym..

[47] Mao S.Y., Suo S.K., Wang Y.M., Chi C.F., Wang B. (2024). Systematical Investigation on Anti-Fatigue Function and Underlying Mechanism of High Fischer Ratio Oligopeptides from Antarctic Krill on Exercise-Induced Fatigue in Mice. Mar. Drugs.

[48] Wan J.J., Qin Z., Wang P.Y., Sun Y., Liu X. (2017). Muscle fatigue: General understanding and treatment. Exp. Mol. Med..

[49] Ament W., Verkerke G.J. (2009). Exercise and fatigue. Sports Med..

[50] Wang P.X., Wang D.H., Hu J.M., Tan B.K., Zhang Y., Lin S.L. (2021). Natural bioactive peptides to beat exercise-induced fatigue: A review. Food Biosci..

