# Supplementary material for: The Effect of Whey Peptides and Micronutrients on Improving Exercise Performance in Mice
Source: Nutrients. 2026 Jan 12;18(2):237. doi: 10.3390/nu18020237 (PMC12845258; doi:10.3390/nu18020237)
Supplement: Supplementary file 1 [file nutrients-18-00237-s001.zip › nutrients-4044187-supplementary.pdf]

Contents of Supplementary Materials

SECTION S1 1
SECTION S2 1
SECTION S3 2

Section S1- Paper Spray Ionization Mass Spectrometry (PSI-MS) Analysis of whey peptides

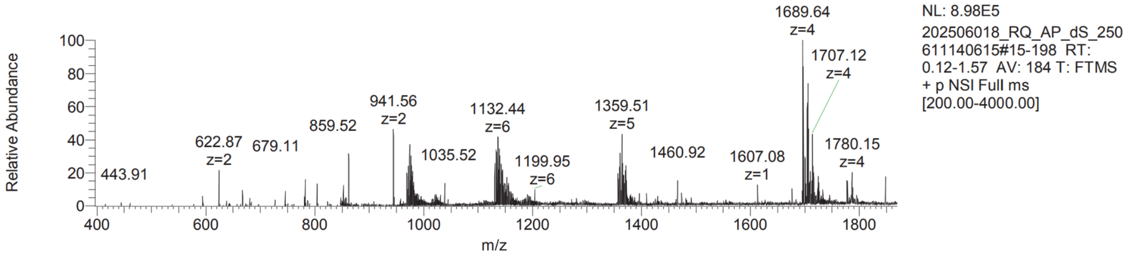

Supplemental Figure S1. Paper Spray Ionization Mass Spectrometry (PSI-MS) Analysis of whey peptides.

Section S2 - Food intake in mice receiving whey peptides and/or micronutrients

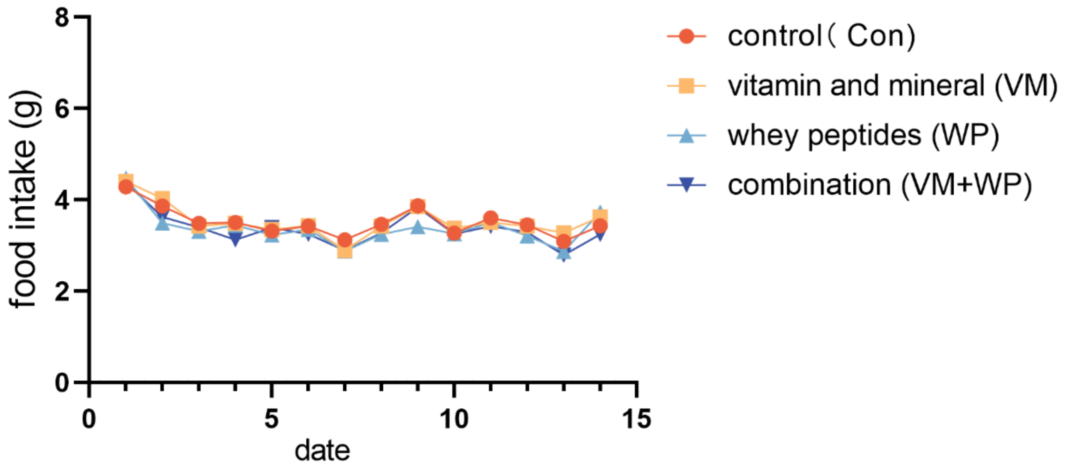

Supplemental Figure S2. Food intake in mice receiving whey peptides and/or micronutrients.

Section S3 - Differential expressed genes in skeletal muscle tissues from mice in four groups (three mice in each group)

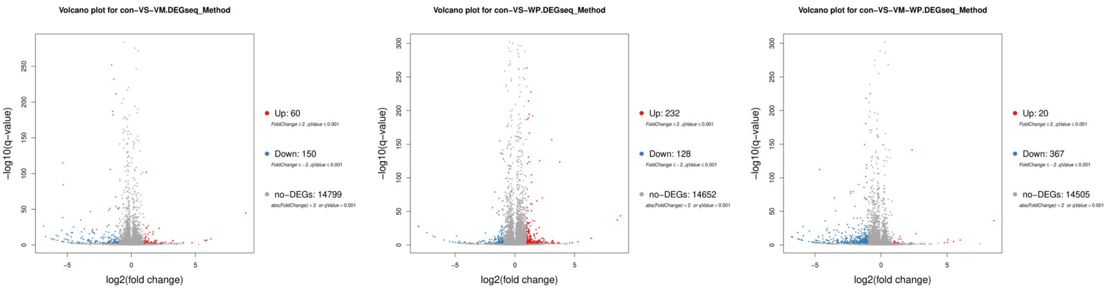

Supplemental Figure S3. Differential expressed genes in skeletal muscle tissues from mice in four groups (three mice in each group).
